# Frailty Syndrome in Non-geriatric Dialysis Patients in Georgia: Prevalence and Clinical Correlates in a Cross-Sectional Study

**DOI:** 10.7759/cureus.114700

**Published:** 2026-08-17

**Authors:** Arwa Fareah Ansar, Nino Tsertsvadze, Barbare Kashibadze, Tasnim Tabassum Taundra, Vasanthapriya Jeevanandam, Mariam Giuashvili, Irma Tchokhonelidze

**Affiliations:** 1 Department of Nephrology, Tbilisi State Medical University, Tbilisi, GEO; 2 Department of Nephrology, Ingorokva High Medical Technology University Clinic, Tbilisi, GEO

**Keywords:** chronic kidney disease (ckd), cross-sectional study, dialysis, frail scale, frailty syndrome, non-geriatric, prevalence

## Abstract

Background

Frailty is a multidimensional syndrome strongly associated with poor quality of life, hospitalization, and mortality in patients with chronic kidney disease (CKD). While frailty in dialysis populations has been well characterized in geriatric patients, its prevalence and correlates among non-geriatric adults remain poorly understood.

Methods

This cross-sectional study enrolled 50 non-geriatric patients (aged 18-64 years) with CKD stage 5D (dialysis-dependent) receiving maintenance hemodialysis at a single center in Tbilisi, Georgia. Frailty was assessed using the FRAIL (Fatigue, Resistance, Ambulation, Illness, and Loss of weight) scale, classifying participants as robust, prefrail, or frail. Demographic data, body mass index (BMI), smoking status, comorbidities, and biochemical correlates were evaluated using Fisher's exact test and multinomial logistic regression.

Results

The mean age was 54.34 ± 8.26 years, and 33 (66%) participants were male. Prefrailty was the most common classification, affecting 22 (44%) participants, followed by robust status in 18 (36%) and frailty in 10 (20%). In multinomial regression, smoking history (pack-years) was inversely associated with frailty in both the frail-vs-robust (p = 0.021) and prefrail-vs-frail (p = 0.005) comparisons. Phosphate levels were significantly associated with frailty in the prefrail-vs-frail comparison (p = 0.04) and showed a non-significant trend in the frail-vs-robust comparison (p = 0.092). Dialysis duration was significantly associated with frailty in the prefrail-vs-frail comparison only (p = 0.016). A non-linear relationship between BMI and frailty was observed, with the highest frailty prevalence among participants with class I obesity, affecting four (57.14%) participants of that subgroup. Comorbidities showed no statistically significant associations with frailty status.

Conclusion

Frailty and prefrailty are common among non-geriatric patients with CKD stage 5D, indicating that frailty in this population is not solely age-driven. Phosphate levels and dialysis duration emerged as clinically relevant correlates, supporting the incorporation of routine frailty screening in non-geriatric dialysis care. Larger longitudinal multi-center studies are needed to confirm these findings and clarify their prognostic significance.

## Introduction

Chronic kidney disease (CKD) is a significant global health challenge, with its prevalence steadily increasing due to the growing burden of diabetes, hypertension, and aging populations. In 2023, an estimated 788 million people aged 20 years and older were living with CKD across all stages, corresponding to a global age-standardized prevalence of 14.2% [1]. Among the many complications faced by CKD patients, frailty, a multidimensional syndrome marked by diminished physiological reserve, is of concern given its strong association with poor quality of life, increased hospitalization, and higher mortality [2]. Frailty is especially prevalent among patients with end-stage kidney disease (ESKD) on dialysis, with global estimates ranging from 30% to 80% in hemodialysis (HD) and 65% to 72% in peritoneal dialysis (PD) populations [3].

Age is consistently identified as a key risk factor for frailty in advanced CKD [4-7], and much of the existing literature has understandably focused on geriatric dialysis populations. However, this has left a notable gap: the prevalence and clinical implications of frailty among non-geriatric patients with CKD on dialysis remain poorly characterized. This represents a critical gap, as younger dialysis patients may develop frailty through distinct pathways, driven less by age itself but rather by the cumulative burden of comorbidities, dialysis vintage, and disease severity. They may face different functional, psychosocial, and prognostic consequences than their older counterparts [8]. Characterizing frailty in this subgroup is essential to disentangling its relationship with CKD stage 5 receiving maintenance dialysis (CKD stage 5D) independent of chronological age.

This study aimed to estimate the prevalence of frailty and prefrailty among non-geriatric patients (aged 18-64 years) with CKD stage 5D in Georgia, and to identify the demographic, behavioral, and biochemical correlates of frailty status. Given Georgia's distinct sociodemographic and healthcare landscape, this cohort offers a valuable point of comparison with regional studies, contributing to a broader understanding of frailty within the Caucasus health environment [5,9]. We hypothesized that, within this younger cohort, modifiable risk factors, particularly behavioral and biochemical correlates, would be more strongly associated with frailty than chronological age.

## Materials and methods

Study population

This single-center, cross-sectional study was conducted at Ingorokva High Technology Medical Centre (HTMC), the largest dialysis center in Tbilisi, Georgia. Prior to data collection, hospital records were reviewed to identify all patients aged 18 to 64 years with a confirmed diagnosis of CKD stage 3 or higher, as defined by Kidney Disease: Improving Global Outcomes (KDIGO) staging criteria [10]. A total of 234 patients met this criterion, of whom 130 met criteria for CKD stage 5D (dialysis-dependent). From this eligible pool, patients were consecutively approached for enrollment as they presented for hemodialysis sessions between September 2024 and January 2025.

Inclusion and exclusion criteria

Inclusion criteria comprised age 18 to 64 years, confirmed CKD stage 5D, receipt of maintenance hemodialysis for a minimum duration of three months, and the cognitive capacity to provide informed consent. Exclusion criteria included acute illness or hospitalization within the preceding month, recent acute coronary syndrome (ACS) or cerebrovascular event (within the preceding month), pregnancy or lactation, and chronic debilitating neuromuscular or musculoskeletal disease or dependency in activities of daily living.

Sample size

Of the 130 eligible CKD stage 5D patients identified, 50 were ultimately enrolled and interviewed. This number reflects logistical constraints inherent to the study population and design rather than a pre-specified power calculation. Many patients traveled from distant cities for dialysis and did not consistently adhere to fixed appointment schedules, making them difficult to reach within the study period. Data collection was additionally constrained by the availability of a small team trained to administer the structured interviews. As such, the final pool represents a convenience sample of all eligible patients who could feasibly be enrolled.

Demographic and clinical data

Demographic, clinical, and laboratory data were collected via structured interview and chart review. Self-reported comorbidity history (hypertension, type 2 diabetes mellitus, peripheral artery disease, etc.) was cross-checked against hospital medical records for verification. Key variables included age, sex, body mass index (BMI, kg/m²), BMI category (Centers for Disease Control and Prevention standard cutoffs: underweight <18.5, normal 18.5-24.9, overweight 25-29.9, class I obesity 30-34.9, class II obesity 35-39.9, class III obesity ≥40), smoking status (current, former, never), smoking pack-years, and pack-year classification (never smokers: 0.0 pack-years, light smokers 0.1-20.0 pack-years, moderate smokers 20.1-40.0 pack-years, heavy smokers >40 pack-years) [11]. CKD stage, duration on dialysis (years), and comorbidities were recorded.

Laboratory assessments drawn at the time of interview included serum potassium (K⁺; normal range: 3.5-5.0 mmol/L), bicarbonate (HCO₃⁻; normal range: 21-26 mmol/L in males, 22-26 mmol/L in females), total calcium (Ca²⁺; normal range: 2.1-2.55 mmol/L), and phosphate (normal range: 0.8-1.4 mmol/L), reflecting electrolyte and acid-base status. Complete blood count parameters included red blood cell count (RBC; normal range: 4.44-5.61 ×10¹²/L in males, 3.92-5.08 ×10¹²/L in females), hemoglobin (Hb; normal: >13 g/dL in males, >12 g/dL in females), hematocrit (HCT; normal range: 40-49.4% in males, 36.6-44% in females), and mean corpuscular volume (MCV; normal range: 80-100 fL). Anemia was defined as hemoglobin below the sex-specific normal threshold, and normocytic anemia was defined as anemia with MCV within the normal range.

Ethical approval

This study was approved by the Biomedical Research Ethics Committee of Tbilisi State Medical University (TSMU) (approval number: 5-2024/112). Verbal informed consent was obtained from all participants prior to enrollment, in accordance with the approved study protocol.

This approach was considered appropriate given the minimal-risk, non-interventional nature of the study. Data collection consisted of a structured interview, along with a review of laboratory values already obtained as part of the patients’ routine care. Patients were approached during their regular hemodialysis sessions, and verbal consent allowed them to participate without adding an additional administrative burden during treatment. All data collected were de-identified before analysis to protect confidentiality.

Frailty assessment

We employed the FRAIL (Fatigue, Resistance, Ambulation, Illness, and Loss of weight) scale [12], which provides an overall assessment of an individual patient's frailty status. The FRAIL scale, a validated screening tool, is composed of five domains: fatigue, resistance, ambulation, illness, and unintentional weight loss. Each domain was scored as either 0 (absence) or 1 (presence), yielding a total score ranging from 0 to 5. Fatigue was defined as self-reported exhaustion or feeling tired most or all of the time. Resistance was assessed by the inability to climb 10 steps without resting, while ambulation was evaluated based on the difficulty of walking a few hundred meters independently. The illness domain was determined by the presence of five or more chronic medical conditions. Finally, weight loss was defined as an unintentional reduction of more than 5% of body weight in the past year. Based on the total score, participants were categorized as robust (0 points), prefrail (1-2 points), or frail (3-5 points). The FRAIL scale was administered through structured interviews conducted by trained personnel. This tool has been validated for assessing frailty risk in various clinical and research settings, including among patients with CKD [13].

Statistical analysis

Statistical analyses were performed using Python version 3.11 (Python Software Foundation, Wilmington, Delaware). Continuous variables (age, BMI, dialysis duration, and laboratory parameters) were summarized as mean ± standard deviation (SD). Categorical variables, including sex, BMI category, smoking status, pack-year classification, comorbidities, dialysis duration, and anemia classification, were summarized as frequencies and percentages and reported descriptively across frailty categories (robust, prefrail, frail). Associations between frailty status and comorbidities (hypertension, ischemic heart disease, peripheral arterial disease, type 2 diabetes mellitus) were assessed using Fisher's exact test, comparing the robust group against a combined prefrail/frail group. Multinomial logistic regression was used to evaluate associations between age, dialysis duration, smoking pack-years, and electrolyte parameters (potassium, bicarbonate, calcium, phosphate), and frailty category, with two pairwise comparisons: frail vs. robust, and prefrail vs. frail. Results are reported as regression coefficients with standard errors, z-values, p-values, and 95% confidence intervals (CI). A p-value <0.05 was considered statistically significant. Data were visualized using stacked bar graphs where appropriate.

## Results

Characteristics of the study population

Table 1 summarizes the baseline patient characteristics of the study population. The mean age of the participants was 54.34 ± 8.26 years (range: 18-64 years), and the cohort was predominantly male, comprising 33 (66%) participants. The mean BMI was 26.99 ± 6.03 kg/m², with the largest proportion of participants classified as overweight at 20 (40%), followed by normal weight at 16 (32%) and obesity across Class I, II, and III combined at 12 (24%) (comprising seven (14%) in Class I, two (4%) in Class II, and three (6%) in Class III). Regarding smoking history, 23 (46%) of the participants were non-smokers, 13 (26%) were former smokers, and 14 (28%) were current smokers, with a mean smoking history of 31.69 ± 32.28 pack-years. Patients had a mean dialysis duration of 9.81 ± 7.21 years, with 22 (44%) having been on dialysis for more than 10 years. The most common comorbidity was hypertension (HTN), affecting 45 (90%) participants, followed by peripheral artery disease (PAD) in 13 (26%), ischemic heart disease (IHD) in 12 (24%), and type 2 diabetes mellitus (T2DM) in 12 (24%). Additional comorbidities, including congestive heart failure (CHF), asthma, chronic obstructive pulmonary disease (COPD), type 1 diabetes mellitus (T1DM), thyroid disease, arthritis, prior stroke, liver disease, and systemic lupus erythematosus (SLE), are detailed in Table 1.

**Table 1:** Characteristics of the study population. BMI: body mass index; HTN: hypertension; IHD: ischemic heart disease; CHF: congestive heart failure; PAD: peripheral artery disease; COPD: chronic obstructive pulmonary disease; T2DM: type 2 diabetes mellitus; T1DM: type 1 diabetes mellitus; SLE: systemic lupus erythematosus. BMI categories based on CDC standard cutoffs: underweight = <18.5 kg/m²; normal = 18.5-24.9 kg/m²; overweight = 25-29.9 kg/m²; Class I obesity = 30-34.9 kg/m²; Class II obesity = 35-39.9 kg/m²; Class III obesity = ≥40 kg/m². Pack-year classification: light smoker = 0.1-20.0 pack-years; moderate smoker = 20.1-40.0 pack-years; heavy smoker = >40 pack-years. * Thyroid disease includes hypothyroidism, hyperthyroidism, and other unspecified thyroid disorders. Arthritis includes inflammatory and non-inflammatory types, but specific classifications were not available. Liver disease includes cirrhosis, liver failure, and other hepatic conditions.

Patient characteristics	n (%)
Male	33 (66)
Female	17 (34)
Mean age (years)	54.34 ± 8.26
18-40 years	3 (6)
41-50 years	9 (18)
51-60 years	28 (56)
61-64 years	10 (20)
Mean BMI (kg/m²)	26.99 ± 6.03
Underweight	2 (4)
Normal	16 (32)
Overweight	20 (40)
Obesity (Class I)	7 (14)
Obesity (Class II)	2 (4)
Obesity (Class III)	3 (6)
Smoking status	
Non-smoker	23 (46)
Former smoker	13 (26)
Current smoker	14 (28)
Mean pack-years	31.69 ± 32.28
Light smoker	13 (48.10)
Moderate smoker	8 (29.70)
Heavy smoker	6 (22.20)
Duration on dialysis	
Mean duration on dialysis (years)	9.81 ± 7.21
0-1 years	3 (6)
1-5 years	14 (28)
5-10 years	11 (22)
>10 years	22 (44)
Comorbidities	
HTN	45 (90)
IHD	12 (24)
CHF	5 (10)
PAD	13 (26)
Asthma	8 (16)
COPD	4 (8)
T2DM	12 (24)
T1DM	2 (4)
Thyroid disease*	4 (8)
Arthritis*	9 (18)
History of stroke	3 (6)
Liver disease*	3 (6)
SLE	2 (4)

Laboratory data of the study participants

Table 2 presents the laboratory data of the study participants. Electrolyte disturbances were common with 38 (76%) of the participants having low bicarbonate (<22 mmol/L; mean = 20.5 ± 2.34 mmol/L), 20 (40%) having hyperkalemia (potassium >5.0 mmol/L; mean = 4.77 ± 0.80 mmol/L), 25 (50%) having hypocalcemia (total calcium <2.1 mmol/L; mean = 2.07 ± 0.25 mmol/L), and 35 (70%) having elevated phosphate levels (>1.4 mmol/L; mean = 1.72 ± 0.47 mmol/L). Anemia was prevalent, affecting 30 (60%) male participants (hemoglobin <13 g/dL) and 14 (28%) female participants (hemoglobin <12 g/dL). The majority of anemic participants fell into the normocytic category, at 38 (76%) (mean hemoglobin = 10.59 ± 1.75 g/dL).

**Table 2:** Laboratory data of the study participants. CBC: complete blood count; MCV: mean corpuscular volume; RBC: red blood cell count.

Laboratory data	n (%)
Electrolytes	
Mean bicarbonate (mmol/L)	20.5 ± 2.34
Bicarbonate < 22 (mmol/L)	38 (76)
Mean potassium (mmol/L)	4.77 ± 0.80
Potassium > 5.0 (mmol/L)	20 (40)
Mean total calcium (mmol/L)	2.07 ± 0.25
Calcium < 2.1 (mmol/L)	25 (50)
Mean phosphate (mmol/L)	1.72 ± 0.47
Phosphate > 1.4 (mmol/L)	35 (70)
CBC	
Mean RBC (10^12^/L)	3.53 ± 0.65
Mean hemoglobin (g/dl)	10.59 ± 1.75
Mean hematocrit (%)	31.54 ± 6.81
Mean MCV (fL)	91.31 ± 6.53
Anemia assessment	
Males with hemoglobin <13 (g/dL)	30 (60)
Females with hemoglobin <12 (g/dL)	14 (28)
Anemia classification	
Microcytic (MCV < 80 fL)	3 (6)
Normocytic (MCV 80–100 fL)	38 (76)
Macrocytic (MCV > 100 fL)	3 (6)

Frailty distribution

Table 3 reports the prevalence of frailty in our study population. Among the 50 participants, 18 (36%) were classified as robust (non-frail), 22 (44%) as prefrail, and 10 (20%) as frail. We assessed the distribution of frailty across key demographic and clinical characteristics of the study population, including age, sex, BMI, smoking status, dialysis duration, anemia status, and comorbidities.

**Table 3:** Prevalence of frailty.

Frailty	n (%)
Robust (non-frail)	18 (36)
Prefrail	22 (44)
Frail	10 (20)

Frailty vs. Age Groups

**Figure 1:**
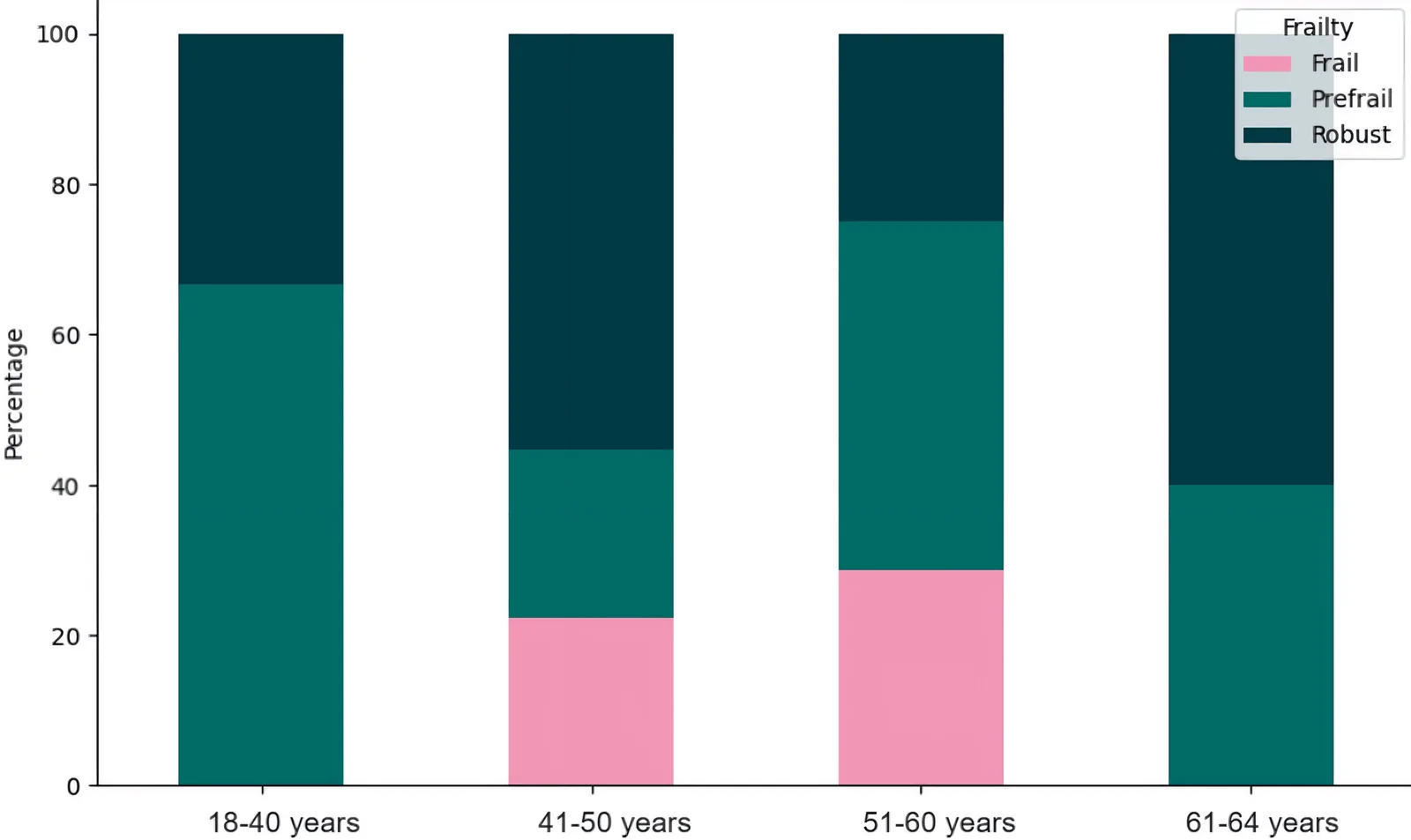
Distribution of frailty categories (robust, prefrail, and frail) across age groups. The x-axis represents age (in years), while the y-axis shows the percentage of participants within each frailty category for each age group.

The distribution of frailty categories across age groups is represented in Figure 1. Among participants aged 18-40 years, two (66.7%) were prefrail, and one (33.3%) was robust, with no participants classified as frail. In the 41-50 age group, four (44.44%) were robust, three (33.33%) were prefrail, and two (22.22%) were frail. Frailty prevalence was highest in the 51-60 age group, with 13 (46.43%) prefrail, eight (28.57%) frail, and seven (25%) robust participants. Among participants aged 61-64 years, six (60%) were robust, and four (40%) were prefrail, with no participants classified as frail.

Frailty vs. Sex

**Figure 2:**
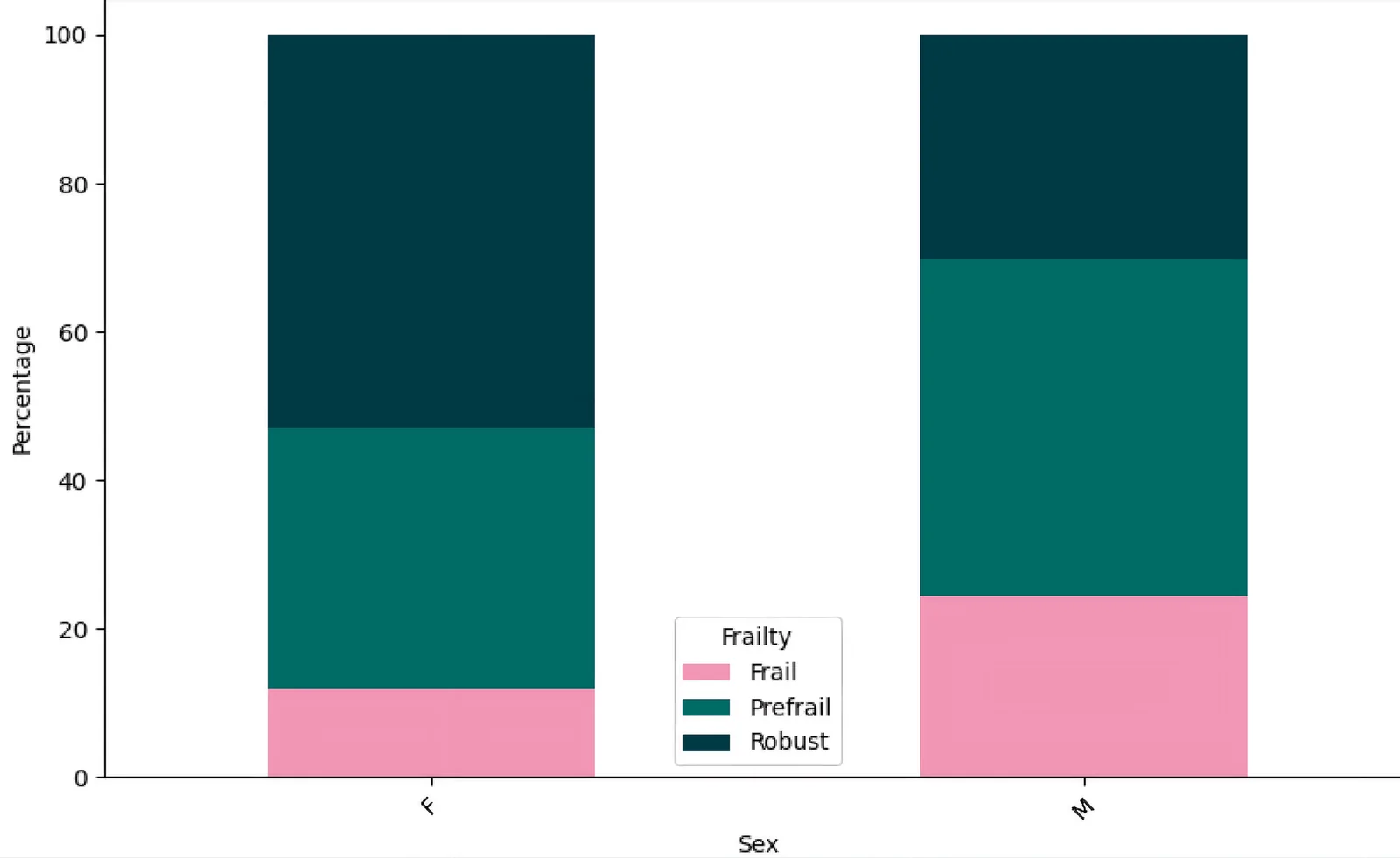
Distribution of frailty categories (robust, prefrail, and frail) among female and male participants. The x-axis represents sex (F: female, M: male), while the y-axis shows the percentage of participants within each frailty category for both sexes.

Figure 2 shows the distribution of frailty categories by sex. Among male participants, 10 (30.3%) were robust, 15 (45.45%) were prefrail, and eight (24.24%) were frail. Among female participants, eight (47.06%) were robust, seven (41.18%) were prefrail, and two (11.76%) were frail.

Frailty vs. BMI Category

**Figure 3:**
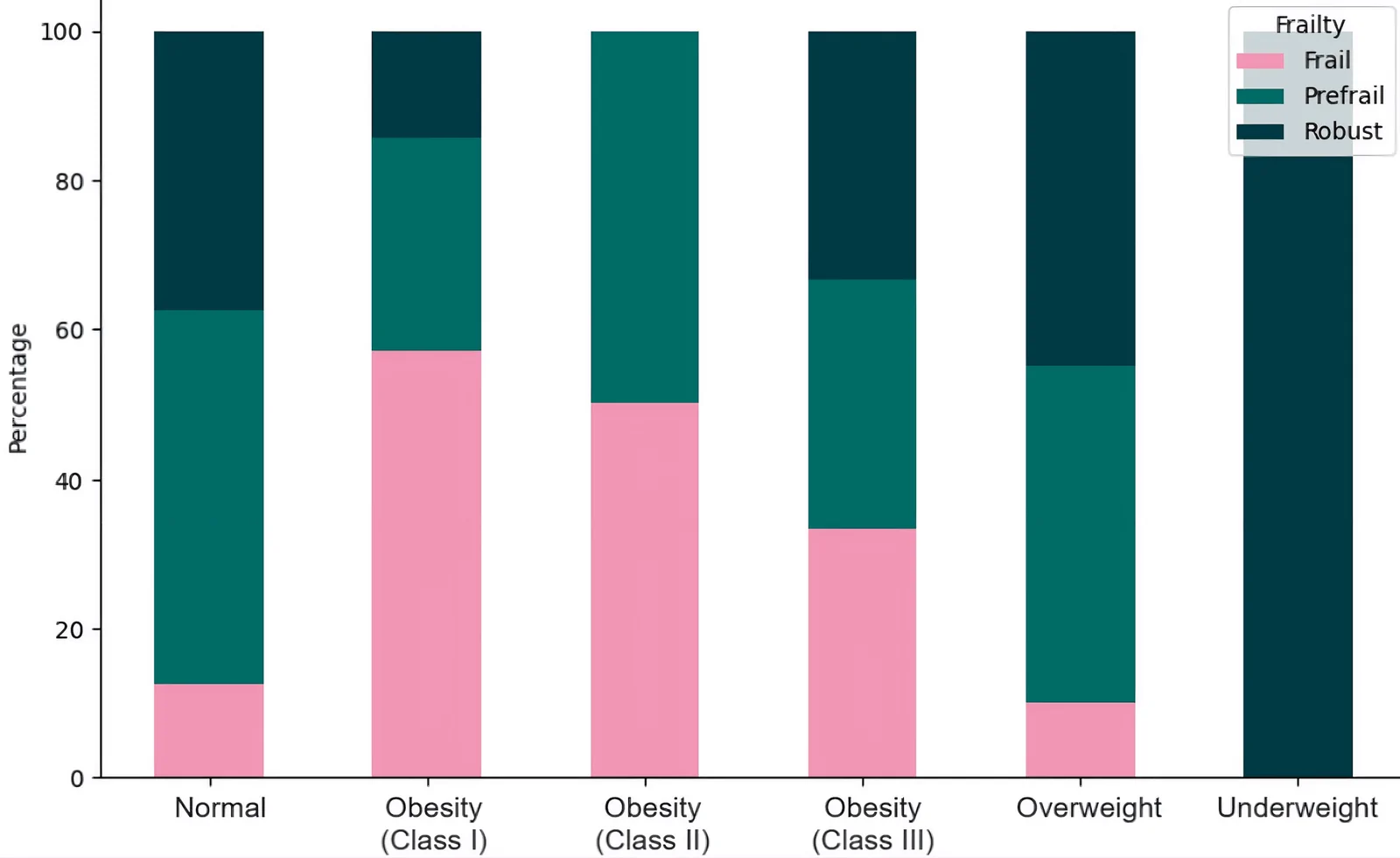
Distribution of frailty categories (robust, prefrail, and frail) across different BMI categories. The x-axis represents BMI categories, while the y-axis shows the percentage of participants within each frailty group for each BMI category.

Figure 3 shows the distribution of frailty categories across different BMI categories. All the underweight participants were robust, at two (100%). Among normal-weight participants, eight (50%) were prefrail, six (37.5%) were robust, and two (12.5%) were frail. Overweight participants were evenly split between robust and prefrail, at nine (45%) each, with two (10%) frail participants. Among obesity class I participants, more than half were frail, at four (57.14%), followed by prefrail at two (28.57%) and robust at one (14.29%). Obesity class II participants were evenly split between prefrail and frail, at one (50%) each, and obesity class III participants were evenly distributed across all three frailty categories, at one (33.3%) each.

Frailty vs. Smoking Status

**Figure 4:**
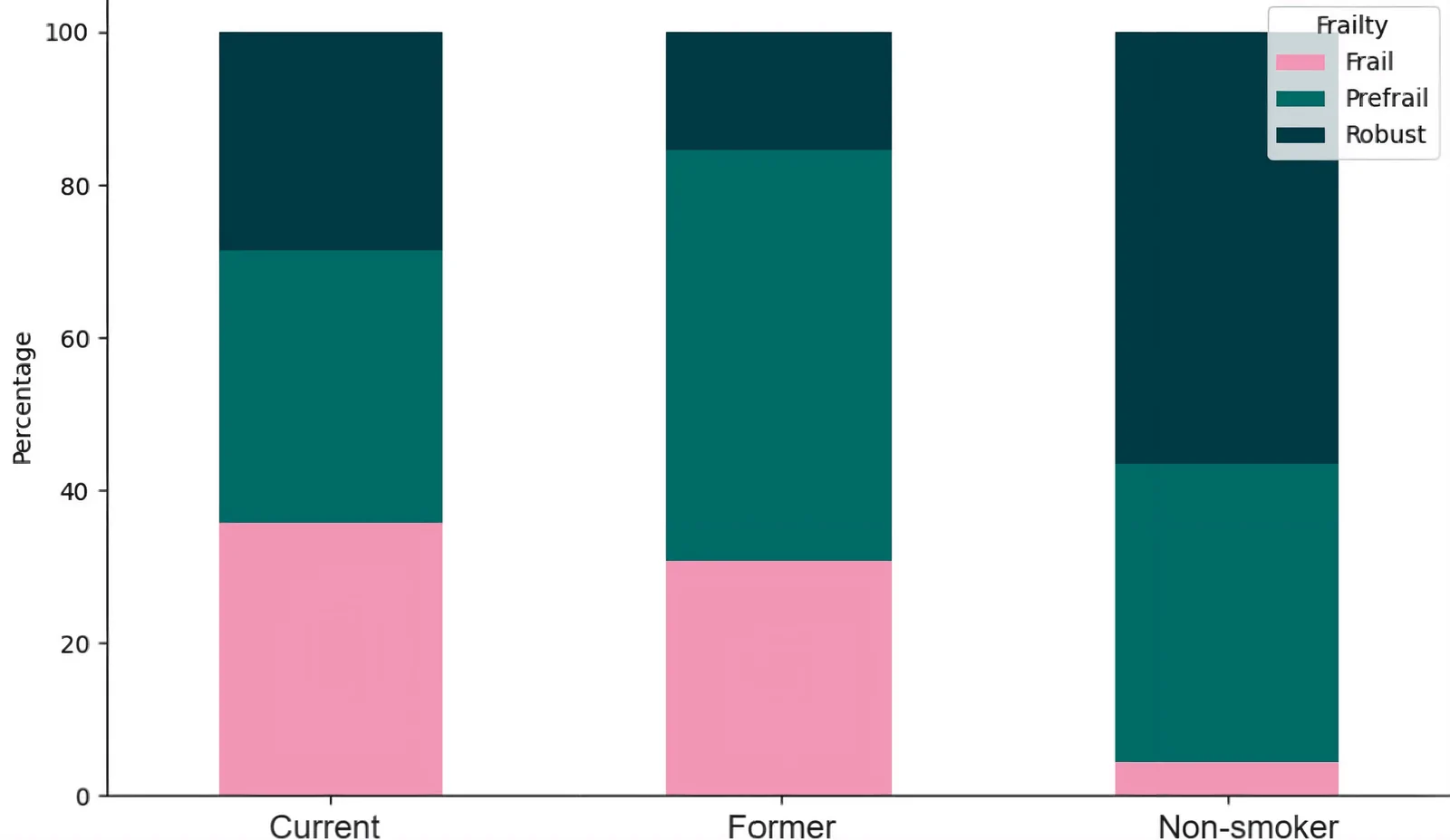
Distribution of frailty categories (robust, prefrail, and frail) across smoking history. The x-axis represents smoking history categories, while the y-axis shows the percentage of participants within each frailty category for each smoking status group.

Figure 4 shows the distribution of frailty categories across smoking history. Among non-smokers, more than half were robust at 12 (52.17%), followed by prefrail at 10 (43.47%) and frail at one (4.3%). Current smokers were evenly split between prefrail and frail at five (35.7%) each, with four (28.5%) robust. Among former smokers, more than half were prefrail at seven (53.85%), followed by frail at four (30.77%) and robust at two (15.38%).

Frailty vs. Dialysis Duration

**Figure 5:**
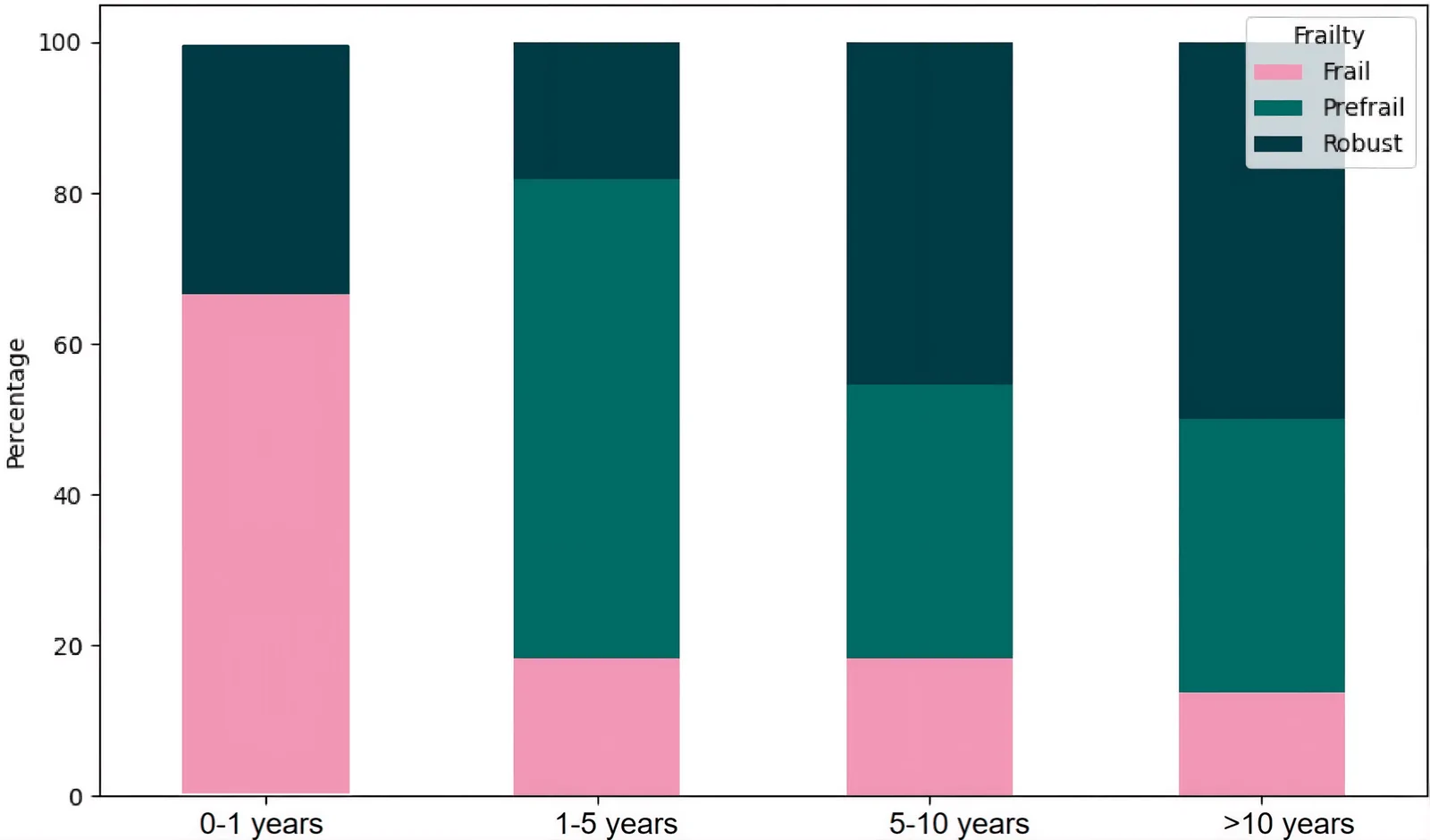
Distribution of frailty categories (robust, prefrail, and frail) across dialysis duration. The x-axis represents the duration of dialysis (in years), while the y-axis shows the percentage of participants within each frailty category for each duration group.

Figure 5 shows the distribution of frailty categories across dialysis duration. Among participants on dialysis for one to two years, two (66.7%) were frail, and one (33.3%) was robust, with no participants classified as prefrail. Among those on dialysis for one to five years, the majority were prefrail at nine (64.29%), followed by frail at three (21.43%) and robust at two (14.29%). Participants on dialysis for five to 10 years were predominantly robust at five (45.45%), followed by prefrail at four (36.36%) and frail at two (18.18%). Participants on dialysis for more than 10 years were also predominantly robust at 10 (45.45%), followed by prefrail at nine (40.91%) and frail at three (13.64%).

Frailty vs. Anemia Status

**Figure 6:**
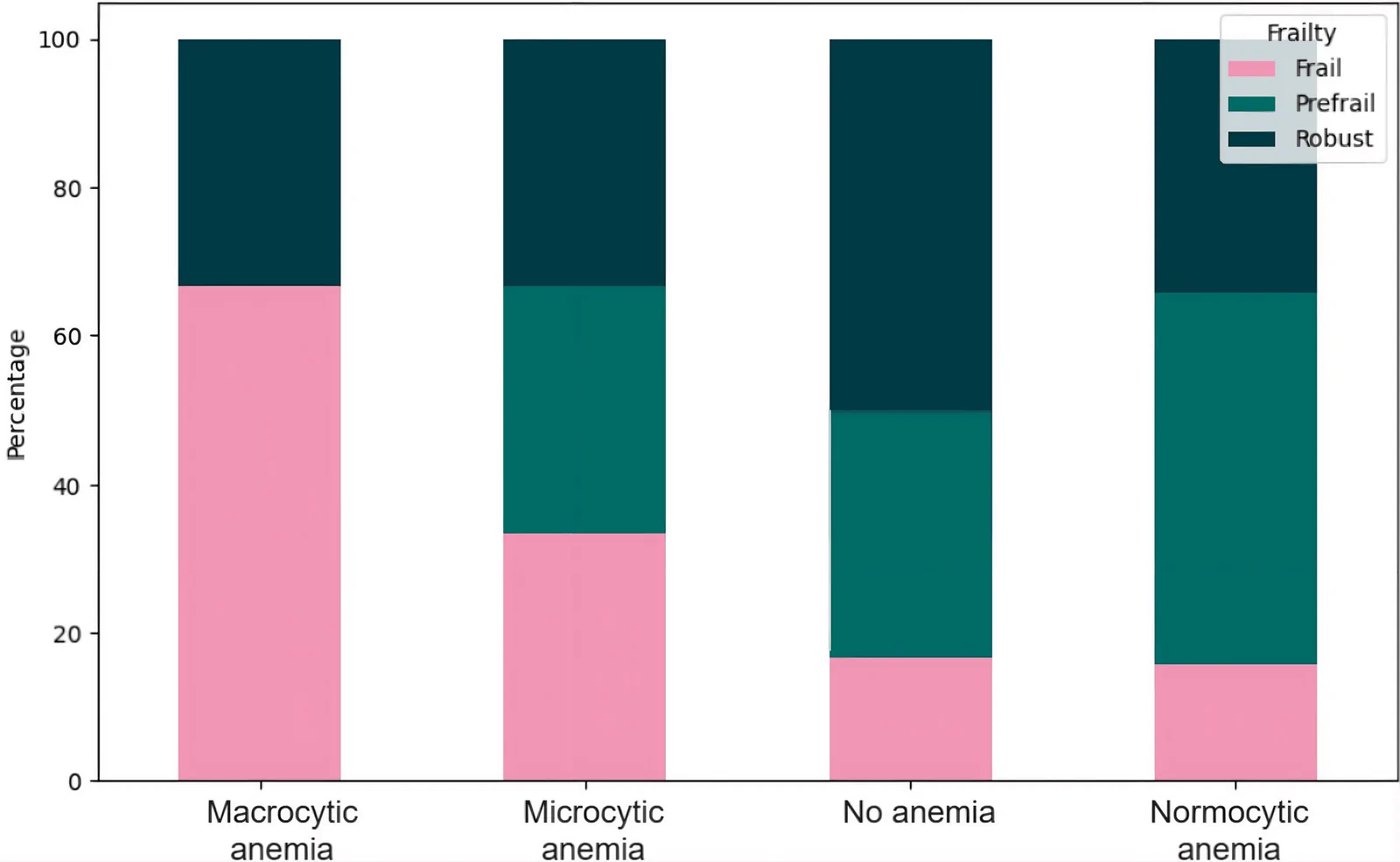
Distribution of frailty categories (robust, prefrail, and frail) across anemia status. The x-axis represents anemia status, while the y-axis shows the percentage of participants within each frailty category for each anemia status group.

Figure 6 shows the distribution of frailty categories across anemia status. Among participants with no anemia, three (50%) were robust, two (33%) were prefrail, and one (17%) was frail. Participants with microcytic anemia were evenly distributed across all three categories at one (33.3%) each. Participants with macrocytic anemia were predominantly frail at two (66.7%), with the remainder frail at one (33.3%), and no participants were classified as robust. Among participants with normocytic anemia, 19 (50%) were prefrail, 13 (34.2%) were robust, and six (15.8%) were frail.

Frailty vs. Comorbidities

Table 4 compares comorbidity prevalence between the combined prefrail/frail group and the robust group. HTN was similarly prevalent in both groups, at 16 (88.9%) in the robust group versus 29 (90.6%) in the prefrail/frail group (p > 0.9999). IHD was more common in the prefrail/frail group at 10 (31.25%) than in the robust group at two (11.1%), though this difference did not reach statistical significance (p = 0.1702). PAD was similarly more prevalent in the prefrail/frail group at nine (28.13%) versus four (22.22%) in the robust group (p = 0.746), as was T2DM at nine (28.13%) prefrail/frail versus three (16.67%) robust (p = 0.4973). No statistically significant associations were found between frailty status and any assessed comorbidity.

**Table 4:** Summary of frailty prevalence against most common comorbidities in participants. HTN: hypertension; IHD: ischemic heart disease; PAD: peripheral artery disease; T2DM: type 2 diabetes mellitus. All comparisons were assessed using Fisher’s exact test. Statistically significant values are indicated by a p-value of less than 0.05.

Comorbidity	Robust, n (%)	Prefrail + Frail, n (%)	p-value
HTN	16 (88.9)	29 (90.6)	>0.9999
IHD	2 (11.1)	10 (31.25)	0.1702
PAD	4 (22.22)	9 (28.13)	0.746
T2DM	3 (16.67)	9 (28.13)	0.4973

Multinomial Logistic Regression Analysis

Multinomial logistic regression was conducted to examine associations between frailty status and clinical/demographic variables, comparing frail vs. robust and prefrail vs. frail groups. Independent variables included age, dialysis duration, smoking pack-years, and electrolyte parameters (potassium, bicarbonate, calcium, phosphate). In the frail vs. robust group, pack-years was significantly associated with frail status (coefficient: −3.47, 95% CI: −6.43 to −0.51, p = 0.021), indicating that greater smoking history was associated with lower odds of frailty relative to robustness in this model. Phosphate levels did not reach statistical significance (coefficient: 2.63, 95% CI: −0.43 to 5.69, p = 0.092) (Table 5). In the prefrail vs. frail group, dialysis vintage (coefficient: 1.81, 95% CI: 0.34 to 3.28, p = 0.016), pack-years (coefficient: −4.65, 95% CI: −7.90 to −1.41, p = 0.005), and phosphate levels (coefficient: 3.24, 95% CI: 0.14 to 6.34, p = 0.04) were all significantly associated with frailty status (Table 6).

**Table 5:** Multinomial logistic regression analysis: frail vs. robust. This table presents the results of a multinomial logistic regression analysis comparing frail versus robust individuals. For each variable, the coefficient, standard error, z-value, p-value, and 95% confidence interval are provided. Statistically significant results are indicated by p-values less than 0.05.

Variable	Coefficient	Standard error	z-value	p-value	95% confidence interval
Constant (intercept)	2.5329	1.092	2.319	0.02	0.392 - 4.674
Age (years)	-0.5087	0.709	-0.718	0.473	(-1.897) - 0.88
Duration on dialysis (years)	1.108	0.686	1.615	0.106	(-0.237) - 2.453
Pack-years	-3.4698	1.509	-2.299	0.021	(-6.428) - (-0.512)
K+ (potassium) (mmol/L)	-0.3501	0.572	-0.612	0.541	(-1.472) - 0.772
HCO3- (bicarbonate) (mmol/L)	-0.7617	0.676	-1.127	0.26	(-2.087) - 0.563
Ca2+ total (calcium) (mmol/L)	0.5661	0.539	1.049	0.294	(-0.491) - 1.623
Phosphate (mmol/L)	2.6287	1.561	1.684	0.092	(-0.431) - 5.688

**Table 6:** Multinomial logistic regression analysis: prefrail vs. frail. This table presents the results of a multinomial logistic regression analysis comparing prefrail versus frail individuals. For each variable, the coefficient, standard error, z-value, p-value, and 95% confidence interval are provided. Statistically significant results are indicated by p-values less than 0.05

Variable	Coefficient	Standard error	z-value	p-value	95% confidence interval
Constant (intercept)	2.0407	1.111	1.836	0.066	(-0.137) - 4.219
Age (years)	-0.7463	0.714	-1.046	0.296	(-2.145) - 0.653
Duration of dialysis (years)	1.8084	0.75	2.41	0.016	0.338 - 3.279
Pack years	-4.6536	1.657	-2.809	0.005	(-7.901) - (-1.406)
K+ (potassium) (mmol/L)	-1.0288	0.635	-1.62	0.105	(-2.273) - 0.216
HCO3- (bicarbonate) (mmol/L)	-0.2793	0.687	-0.406	0.684	(-1.626) - 1.067
Ca2+ total (calcium) (mmol/L)	0.6589	0.545	1.208	0.227	(-0.41) - 1.728
Phosphate (mmol/L)	3.2425	1.582	2.05	0.04	0.143 - 6.342

## Discussion

This study demonstrates a substantial burden of frailty among non-geriatric patients with CKD stage 5D in Georgia, of whom 22 (44%) met criteria for prefrailty and 10 (20%) for frailty, with only 18 (36%) of the patients classified as robust. This combined prefrailty/frailty burden of 64% falls within the range reported in general dialysis populations, despite our cohort being restricted to non-geriatric adults, suggesting that frailty in this population is not solely an age-driven phenomenon but rather reflects the cumulative physiological burden of advanced CKD and dialysis exposure itself [2,3].

A striking finding in our study was that smoking history (measured in pack-years) was inversely associated with frailty status in both the frail-vs-robust (p = 0.021) and prefrail-vs-frail (p = 0.005) comparisons, implying that participants with greater smoking history had lower odds of being classified as frail. This finding runs contrary to the well-established link between cumulative smoking exposure and vascular and functional decline in the general population [11]. However, a similar inversion of expected risk-factor relationships, termed "reverse epidemiology," has been well documented in dialysis populations, where conventional cardiovascular risk factors are sometimes associated with paradoxically better outcomes [14]. One proposed explanation is survival bias, wherein patients with heavier smoking histories who were also frail may not have survived to reach dialysis or non-geriatric enrollment, leaving a comparatively hardier subgroup of long-term smokers in cross-sectional dialysis cohorts [14]. Unmeasured confounding and our modest sample size, particularly within the frail subgroup (n = 10), may also contribute to this finding, warranting further exploration in larger, longitudinal cohorts.

An interesting pattern emerged in the relationship between BMI and frailty status. Frailty prevalence for overweight participants was two (10%) and rose sharply among participants with obesity class I to four (57.14%), while class II and III showed a prevalence of one (50%) and one (33.33%), respectively. This suggests a non-linear relationship between adiposity and frailty in this cohort. The pattern stands in contrast to the "obesity paradox" often reported in dialysis populations, in which higher BMI is associated with improved survival within the reverse epidemiology framework [14]. Rather than reflecting a protective effect of higher body mass, our findings may point to sarcopenic obesity, a condition in which increased fat mass coexists with reduced muscle mass and strength, an increasingly recognized driver of frailty in CKD [15]. As BMI alone cannot distinguish fat mass from lean mass, it may inadequately capture the muscle wasting that underlies physical frailty in this population, potentially explaining why higher BMI categories were not uniformly protective in our cohort. Given the small subgroup sizes within some BMI categories (e.g., n = 2 for underweight and n = 2 for obesity class II), this comparison was descriptive only and was not included in the regression model. Future studies incorporating direct measures of body composition, such as muscle mass or waist circumference, would help clarify the mechanism underlying this relationship.

Another noteworthy finding was phosphate levels, which showed higher phosphate levels associated with greater odds of frailty. In the frail-vs-robust comparison (p = 0.092), a trend toward association with frailty reached statistical significance in the prefrail-vs-frail comparison (p = 0.04). This is consistent with recent findings that phosphate is independently associated with frailty in patients with CKD [16]. Hyperphosphatemia is a central feature of CKD-mineral and bone disorder (CKD-MBD), which may increase energy expenditure and contribute to malnutrition, inflammation, and muscle wasting, all mechanisms plausibly linking elevated phosphate to frailty [16]. Given that 35 (70%) participants in our cohort had elevated phosphate levels, this association may represent a clinically relevant pathway linking mineral metabolism to frailty in non-geriatric dialysis patients, though confirmation in larger, longitudinal cohorts is needed before treatment implications can be drawn.

Finally, the duration of dialysis showed a distinct pattern across the two comparisons described in our multinomial logistic regression analysis. Dialysis vintage was not significantly associated with frailty in the frail-vs-robust model (p = 0.106), but was significantly associated with increased odds of frailty in the prefrail-vs-frail comparison (p = 0.016). This aligns with findings from a large nationwide Japanese cohort, which reported a progressive, dose-dependent association between longer dialysis vintage and higher frailty prevalence [17]. Our results suggest that cumulative dialysis exposure may play a more prominent role in the transition from prefrailty to frailty than in distinguishing frail from robust status. This finding may potentially reflect a threshold effect in which the physiological toll of prolonged dialysis becomes most apparent once early frailty markers are already present.

Taken together, these findings support our hypothesis that modifiable and disease-related factors, particularly mineral metabolism and dialysis exposure, are meaningfully associated with frailty status in this younger cohort, independent of chronological age. This reinforces the case for viewing frailty in dialysis patients as a multidimensional syndrome driven by disease burden rather than a condition confined to older adults. Routine frailty screening in non-geriatric dialysis populations, coupled with early attention to phosphate control and modifiable risk factors, may help identify at-risk patients before the transition to more severe frailty occurs.

This study's strengths include a relatively balanced distribution across robust, prefrail, and frail categories, which improved between-group comparability. The use of a validated, structured frailty assessment tool alongside comprehensive laboratory and clinical data allowed for a multidimensional statistical evaluation of frailty correlates.

Limitations of the study include the single-center design and modest sample size, which limit statistical power and constrain generalizability beyond this population. The FRAIL scale, while validated and widely used, has limited prior validation, specifically in non-geriatric dialysis populations, and self-reported domains may be subject to social desirability bias, potentially underestimating true frailty prevalence. The cross-sectional design precludes causal inference and does not allow for assessment of frailty progression over time. Unmeasured confounders (including medication adherence, physical activity, and dietary intake) were not captured and may influence the observed associations.

Future research should prioritize longitudinal designs to clarify the temporal relationship between dialysis exposure, mineral metabolism, and frailty progression, and should investigate underlying mechanisms like chronic inflammation, malnutrition, and metabolic dysregulation that may drive frailty development in CKD. Studies incorporating both geriatric and non-geriatric populations across a broader range of disease duration would help clarify whether the correlates identified here are unique to younger dialysis patients or reflect broader CKD-related frailty pathways.

## Conclusions

This study reveals a substantial burden of frailty and prefrailty among non-geriatric patients with CKD stage 5D in Georgia, demonstrating that frailty in dialysis populations is not solely an age-related phenomenon but reflects the broader physiological burden of advanced kidney disease. Phosphate levels and dialysis duration emerged as clinically meaningful correlates of frailty status in this younger cohort, pointing to mineral metabolism and cumulative dialysis exposure as potential targets for early identification and intervention. These findings support incorporating routine frailty screening, such as the FRAIL scale, into the clinical care of non-geriatric dialysis patients, rather than reserving such assessments for older adults alone. Larger longitudinal multi-center studies are needed to confirm these associations and determine whether early frailty identification can meaningfully improve outcomes in this younger dialysis population.
